# Efficacy of Tui Na in idiopathic constipation in children with cerebral palsy: a randomized controlled clinical trial

**DOI:** 10.3389/fped.2024.1503591

**Published:** 2025-01-23

**Authors:** Huijuan Wang, Bichan Chen, Qian Long, Qiuping Yang, Jiawen Mao, Qinghua Ma, Xingqian Yi, Ying Wang, Yinghan Liu, Zhiliang Cao, Jianda Xu, Yong Ye, Wei Tang

**Affiliations:** ^1^ Department of Acupuncture, Moxibustion and Tuina, The First Hospital of Hunan University of Chinese Medicine, Changsha, China; ^2^ Department of Traditional Chinese Medicine, Changsha Hostipal for Maternal & Child Health Care, Changsha, China; ^3^ Department of Pediatrics, Affiliated Hospital of Jiangxi University of Chinese Medicine, Nanchang, Jiangxi, China; ^4^ Department of Orthopedics, Changzhou Hospital Affiliated with Nanjing University of Chinese Medicine, Changzhou, Jiangsu, China

**Keywords:** tuina, idiopathic constipation, children, cerebral palsy, effect

## Abstract

**Objective:**

To investigate the efficacy and potential adverse events of Tuina therapy for idiopathic constipation in children with cerebral palsy (CP).

**Methods:**

A total of 60 CP children with idiopathic constipation were enrolled and randomly divided into Tuina and control groups. The control group was treated with basic treatment and 12 sessions of lactulose oral solution, whereas the Tuina group received basic treatment and 12 sessions of infantile Tuina treatment. The following parameters were compared: the Bristol stool form scale (BSFS), the Constipation Assessment Scale (CAS) and the improvement in constipation. In addition, adverse effects were recorded.

**Results:**

At 4 weeks after the final treatment, the percentage of infants whose constipation improved was 23 (76.7%) in the Tuina group and 21 (70.0%) in the control group (*P* = 0.771). Initially, the CAS score, weekly bowel movements and proportion of infants with bowel evacuation ≥2 h were comparable between the two groups (*P* > 0.05). At 4 weeks after the final treatment, the CAS score, weekly bowel movements and proportion of infants with bowel evacuation ≥2 h all significantly improved (*P* < 0.05) compared with those in the initial situation. However, no difference was found in either group at 4 weeks after the final treatment. No serious adverse reactions (such as diarrhea, abdominal pain, vomiting, subcutaneous redness, skin breakage, or syncope) were recorded.

**Conclusions:**

Tuina was as effective as medical care in addition to basic treatment for both groups. The results of this study suggest that Tuina, as a nonpharmacological therapy, may be helpful as an alternative treatment for constipation. More advanced research and large-sample studies should be conducted.

## Introduction

Cerebral palsy (CP) is a nonprogressive neurologic condition that manifests as movement and muscle tone impairments due to neurological developmental abnormalities. It affects approximately 2.5 per 1,000 births worldwide (1). Children with CP suffer from highly reduced mobility and subsequent constipation. In addition, most children with CP who are constipated may also have other systemic symptoms, such as sleep disturbances (including prolonged sleep onset, shortened sleep duration, and easy awakening) and emotional irritability (2). As the most common problem, with a high incidence rate of 75%, constipation usually results from the intake of several components, including reduced fiber and liquid intake, mobility reduction, damage to the central nervous system, and therapeutic drugs (3–5). In addition, it worsens as the severity of CP increases (3).

To date, no consensus exists on the treatment of constipation in children with CP (6). The treatment mainly focuses on improving the symptoms of constipation, such as lifestyle guidance, pharmacological therapy and nonpharmacological therapy (7). Imanieh M.H. et al. compared three different therapeutic methods and suggested that effective and safe agents are still important treatmen choices in the treatment of chronic constipation in CP children (8). Veugelers R. et al. conducted a cross-sectional observational study and reported that children with severe generalized cerebral palsy often experienced constipation. Laxatives are common medicines, but their dosing is frequently inadequate to prevent symptoms (3). In addition, García Contreras A.A. et al. suggested the use of synbiotics, prebiotics, and probiotics to improve stool characteristics, such as a history of painful defecation (9). However, discomfort associated with anal administration, high drug dependence, and poor long-term efficacy are unavoidable.

As an important alternative and nonpharmacological treatment method to traditional Chinese medicine, Tui Na has shown effective therapeutic effects on constipation (10). However, to our knowledge, few studies have drawn accurate conclusions. The effect of Tui Na on constipation remains controversial and poorly documented.

In the present study, we conducted a randomized controlled clinical trial to investigate the efficacy of Tuina therapy for on idiopathic constipation in children with CP. In addition, we examined the tolerance and safety of Tui Na therapy via adverse effects.

## Materials and methods

This clinical trial was designed and approved by the Ethics Review Committee of The First Hospital of Hunan University of Chinese Medicine (approval number: HN-LL-KY-2022-010-01). Written informed consent was obtained from the parents of all the participants. The study was registered with the China Clinical Trial Registry on April 18, 2022 (registration number: ChiCTR2200058879).

From June 2022 to June 2023, a total of 60 CP children with idiopathic constipation, ranging in age from 16 to 98 (average, 42.70 ± 17.81) months, were enrolled. All the children in the group met the diagnostic criteria for CP and the Rome III criteria for constipation, and no constipation-related therapies were administered in the month before the study (11). All the children were randomly divided into Tuina and control groups (*n* = 30). The control group was subjected to basic treatment and 12 sessions of lactulose oral solution, whereas the Tuina group received basic treatment and 12 sessions of Liu's infantile Tuina treatment. No differences were found in terms of age, sex, body mass index (BMI), duration of disease, or other accompanying conditions. (*P* > 0.05, Table 1).

**Table 1:** Baseline characteristics of all patients.

		Tuina group	Control group	*P* value
Age (m)		49.60 ± 15.06	39.43 ± 22.368	0.281
Gender	Male	21	16	0.288
	Female	9	14	
BMI (kg/m2)		14.67 ± 0.97	14.41 ± 0.95	0.772
Duration(m)		32.93 ± 13.80	26.23 ± 14.83	0.923
Clinical pattern	Tetraplegia	13	14	0.703
	Diplegia	13	11	
	Hemiplegia	4	5	
Stool frequency/wk				
1		18	17	0.822
2		10	11	
3		2	1	
Bristol stool scale				
1		23	22	0.945
2		5	6	
3		2	2	

Children were excluded if they had organic lesions of the rectum or sigmoid colon or congenital anomalies (such as anal stenosis and intestinal stricture), if they had skin breakage or local infection, if they had constipation due to previous surgery, or if their parents were unwilling to participate in the study.

### Therapy regimen

All the children received the same basic treatment for dietary adjustment and increased activity. The Tuina was performed by the same experienced Tuina therapist, who was a major in Tuina.

### Control group

The control group was given a total of 12 sessions of lactulose oral solution (Abbott Biologicals B.V. Netherlands) strictly (3 sessions per week for 4 weeks, with follow-up at 2 weeks and 4 weeks after the end of treatment), and the dose was adjusted according to the age of the child.

### Tuina group

All participants will receive Tuina treatment three times a week (adjusted by initial local condition assessment) and continuously for four weeks. Each Tuina session lasts 20 min. If participants were older than 3 years, 50 times more added for each additional year.

The Tuina regimen includes four steps as follows (all the acupoints are listed in Table 2):

**Table 2:** Tuina acupoints, locations, methods and times.

Acupoints	Location
Tianmen	Located on the face, from the middle of the eyebrows to the front hairline in a straight line
Kangong	Located on the face, from the center of the eyebrow to the tip of the eyebrow in a horizontal line
Taiyang (temple)	Located on the face, in the depression behind the eyebrows
Baihui (DU20)	Located on the head, 5 cun above the middle of the front hairline
Sishencong (EX-HN 1)	Located on the head, 1 cun anterior, posterior and lateral to Baihui,4 acpoints totally
Fengchi (GB 20)	Located on the nape, below the occipital bone, in the depression between the superior sternocleidomastoid muscle and the superior trapezius muscle
Fengfu (GV 16)	Located on the nape, 1 cun directly above the midpoint of the posterior hairline, in the depression below the external occipital protuberance
Yingxiang (LI 20)	Located on the face, 0.5 cun away from the nose, in the nasolabial groove
Zongjin	Located on the palmar side, at the midpoint of the transverse wrist at the back of the palm
Yinyang	Located on both sides of the Zongjin, the side of the little finger is yin and the side of the thumb is yang
Pi jing (spleen meridian)	Located on the threaded surface at the end of the thumb
Gan jing (liver meridian)	Located on the threaded surface at the end of the index finger
Xin jing (heart meridian)	Located on the threaded surface at the end of the middle finger
Fei jing (lung meridian)	Located on the threaded surface at the end of the ring finger
Shen jing (kidney meridian)	Located on the threaded surface at the end of the little finger
Da chang (large intestine)	Located at the radial aspect of the index finger, in a straight line from the tip of the index finger to the first web space
Banmen	Located in the plane of the thenar eminence of the hand
Zhongwan (RN12)	Located on the anterior midline, 4 cun above the umbilicus
Fu (abdomen)	Located in the upper abdomen
Gui Wei	Located at the end of the coccyx
Qi jie gu (seven segmantal bones)	Located in the lumbosacral region, in a straight line from the fourth lumbar vertebra to the end of the coccyx
Ji (spine)	Located on the posterior midline, in a straight line from the first thoracic vertebra to the end of the caudal vertebra
Zusanli (ST 36)	Located in the calf, 3 cun directly below Dubi, and one finger-breadth lateral to the anterior border of the tibia
Jianjing (GB21)	Located on the shoulder, at the midpoint of the line joining Dazhui and acromion

Step 1: The child lay supine or sat while the therapist manipulated the Tianmen acupoint 48 times, parted-pushed the Kangong acupoint 48 times, straight pushed the Taiyang acupoint 48 times, pinched the great tendon 24 times, and broke up Yin Yang (parting-pushing from the central transverse crease of the wrist) 24 times.

Step 2: The child lies supine or sits while the therapist tonifies the spleen meridian 400 times, clears the liver meridian 200 times, tonifies the heart meridian 300 times, then clears the heart meridian 150 times, tonifies the lung meridian 300 times, and tonifies the kidney meridian 400 times.

Step 3: The children lay supine while the therapist kneaded the Baihui acupoint, Si Shencong acupoint, bilateral Fengchi acupoint, and bilateral Yingxiang 100 times each. The therapist then cleared away the Dachang acupoint 150 times, kneaded the Zusanli acupoint 80 times, and kneaded the Tanzhong acupoint and Tiansu acupoint 100 times. The children were subsequently changed to a prone position. The therapist kneaded the Pi Shu acupoint, Shen Shu acupoint, and Dachang Shu acupoint 100 times each. The therapist then manipulated the Qijiegu acupoint 60 times, kneaded the Guiwei acupoint 150 times and pinched the entire spinous process 5 times.

Step 4: The children lied prone or sat while the therapist kneaded Jianjing 2 times.

### Parameters

All assessments were completed by an independent data assessor who was blinded to group allocation. At the initial diagnosis, the researchers would record the child's demographics (name, sex, age, height, weight, and duration), previous diagnostic and treatment history, and complete stool and serum collection. The mothers recorded stool frequency, consistency and the time spent on bowel care each day in a 4-week bowel diary. All follow-up indicators were measured at the initial visit and 4 weeks after the final treatment.

Bristol stool form scale (BSFS) (12): Stool properties are classified into 7 classes: types 4 and 5 are normal stools, whereas types 1, 2, 3, 6, and 7 are abnormal stools. The parents were asked to choose the BSS stool type to represent their children's stools.

Constipation Assessment Scale (CAS): The CAS is used to evaluate the severity of constipation through self-reported items. The total score ranges from 0 (no constipation) to 16 (severe constipation). Parents or caregivers were asked to answer this questionnaire (13).

Constipation improvement was defined as follows: (1) the defecations reached 3 times a week, and the stool consistency had softened (3<BSFS<6); (2) the frequency of stool evacuation attempts (more than 2 h per day) decreased (14).

Adverse effects, such as adverse reactions such as diarrhea, abdominal pain, vomiting, subcutaneous redness, skin breakage, and syncope, were also recorded.

### Sample size calculation

The sample size was estimated via G*Power. On the basis of our preliminary study, a significant difference (5 points) in CAS (alpha 0.05, power 0.80) was detected. Given an expected dropout rate of 10%, at least 30 patients were to be enrolled in each group.

### Statistical analysis

All the data were analyzed via SPSS (version 25.0; IBM Corp, Armonk, NY, USA). The X^2^ test will be used to compare the rates of the count data; the measurement data will be expressed as the means ± standard deviations (x ± s). Independent samples t tests were used for comparisons between groups when normality and equal variances were met; the independent samples Mann‒Whitney U test was used if the overall data did not meet normality. *P* < 0.05 was considered statistically significant.

## Results

At 4 weeks after the final treatment, the percentage of infants with improved constipation was 23 (76.7%) in the Tuina group and 21 (70.0%) in the control group (*P* = 0.771).

Initially, no difference was observed in the CAS score between the treatment groups: 10.47 ± 2.27 vs. 11.76 ± 2.24 (*P* = 0,671, Table 3). The stool frequency in the Tuina group was 17 once per week and 12 twice per week, whereas in the control group it was 16 once per week and 12 twice per week (*P* = 0.550, Table 3). The mean number of weekly bowel movements in both groups was 1.45 ± 0.58 vs. 1.31 ± 0.55 per week. In addition, the number of infants who underwent bowel evacuation >2 h/d was comparable between the two groups (*P* = 0.795, Table 3).

**Table 3:** Comparison of stool frequency and consistency between the tuina group and the control group.

Variables	Tuina group		Control group		*P1* ^a^	*P2* ^b^
	Before	After	Before	After		
Bristol stool scale						
≤2	29	2	28	3	0.550	0.643
≥3	1	28	2	27		
Before/after^c^	<0.001		<0.001			
Bowel movements/wk	1.45 ± 0.58	3.98 ± 0.55	1.31 ± 0.55	3.98 ± 0.65	0.144	0.217
Before/after^c^	<0.001		<0.001			
Bowel evacuation >2 h/d	17	7	18	9	0.795	0.771
Before/after^c^	0.038		0.002			

^a^ Comparison between both groups before treatment.

^b^ Comparison between both groups after treatment.

^c^ Comparison within the same group before and after intervention.

At 4 weeks after the final treatment, the CAS score and bowel evacuation in both groups decreased significantly (*P* < 0.05). The number of weekly bowel movements increased significantly (*P* < 0.001). However, there was no difference in the CAS score or weekly bowel movements between the treatment groups after treatment: 3.63 ± 2.29 vs. 3.76 ± 1.89, 3.98 ± 0.55 vs. 3.98 ± 0.65 (*P* = 0,671, 0.217, respectively; Table 3).

The proportion of infants with more than 2 h per day of painful bowel evacuation decreased significantly in both groups (*P* = 0,671 and 0.217, respectively; Table 3). However, no significant difference was found between the groups 4 weeks after the final treatment.

No serious adverse reactions (such as diarrhea, abdominal pain, vomiting, subcutaneous redness, skin breakage, or syncope) were reported.

## Discussion

In the present study, our findings confirmed that Tuina was an effective therapy for children with CP with constipation. No serious complications were found throughout the entire treatment process.

As a basic therapy, lifestyle guidance emphasizes interventions in the diet, exercise, and bowel habits of the child. Early satiety and poor feeding often occur in approximately 41% of patients with CP, which contributes to undernutrition. To date, there is ongoing debate regarding the recommendation of dietary fiber and fluid intake due to the inadequacy of the study design. The American Society of Pediatric Gastroenterology, Hepatology and Nutrition (NASPGHAN) did not support fiber consumption to prevent constipation (15). However, lower fiber and fluid intake also worsens this condition (4). García Contreras A.A. et al. conducted an analytical cross-sectional study and reported that low fiber and fluid intake and the use of anticonvulsant polytherapy usually resulted in harder stools and lower defecation. They recommended that daily fiber and fluid intake are important for improving constipation (16). At our institute, we still agree with the hypothesis that appropriate fiber and fluid intake can improve constipation. We advised each child with dietary adjustment. Considering the diversity of participants, well-designed high-quality randomized controlled trials are urgently needed to determine the recommendation and appropriate quantity for CP children with constipation.

In children with CP, ambulatory function is often positively correlated with the extent of brain damage. Improving brain function may enhance the brain's ability to regulate the gastrointestinal system, playing a key role in treating constipation in children with CP. Most associations are found between constipation and neurologic diseases and the spine due to damage to the central nervous system. Deterioration of the neurological function of the brain in children with CP is positively correlated with the severity of constipation (17). Disruption of the neural modulation of colonic motility plays a crucial role in the process of constipation (18). Damage to the early central nervous system includes a series of adaptive neuroplasticity processes. Earlier interdisciplinary intervention can improve brain plasticity and improve an individual's condition (19). The acupoints Baihui, Sishencong and Fengchi are pressed to enhance brain function and improve intelligence, which regulate the qi and blood of the Du meridian,and the acupoints Baihui, Sishencong, and Fengchi are pressed to enhance brain function and improve intelligence, regulating the effects of mind regulation and brain development in the theory of traditional Chinese medicine. Our study revealed comparable efficacy between infantile Tuina and oral lactulose treatment for idiopathic constipation in children with CP. At 4 weeks after the final treatment, the percentage of infants with improved constipation was 23 (76.7%) in the Tuina group and 21 (70.0%) in the control group (*P* = 0.771). The tina of the head can promote blood circulation and improve the blood supply to the brain, which partially compensates for damaged brain tissue functions by accelerating the development of the nervous system.

Chronic disability and decreased motor activity due to muscle weakness in the gastrointestinal system are among the most common problems and are associated with the severity of the disorder (20). Deficiencies in abdominal, intestinal, and anal muscle strength may also trigger defecation disorders. A delay in transit time at the entire or segmental colon was associated with constipation in patients with CP. Tuina is a nonpharmacological therapy via performing manipulations on the body surface. The theoretical basis originates from the special meridian-acupoint theory of traditional Chinese medicine. Tuina can induce autonomic responses via cutaneo-visceral reflexes to improve constipation (21).

Children with CP the hypotonic type exhibit delayed motor development and reduced motor activity. Abdominal massage can promote gastrointestinal motility and has a direct therapeutic effect on constipation. In this study, the application of abdominal manipulation was emphasized, and a combination of rubbing, holding, kneading and vibrating techniques was applied directly to the abdomen of the child. The abdomen serves as the body's projection area for the stomach and intestines. External pressure stimulates the intestines, causing deformation or displacement, which promotes intestinal content transmission and facilitates defecation.Moreover, gentle and powerful manipulation of the abdomen can reflexively regulate the central system, reduce the excitability of the descending colon and rectal parasympathetic nerves, increase peristalsis of the descending colon and rectum, and create favorable conditions for defecation (22). The improvement in muscle strength not only increases daily activity and promotes intestinal peristalsis but also enhances the contraction of the abdominal muscles, anal muscles and intestinal wall muscles and increases abdominal pressure, all of which can accelerate fecal excretion and reduce the symptoms of constipation in children. In addition, stretching exercises could reduce spasticity, improve joint mobility, and enhance functional independence. These improvements increase the motility of the gut and relieve constipation by inducing parasympathetic stimulation (23).

## Limitations

There were several limitations in the present study. First, this was a small sample size study, and the follow-up time was only 4 weeks. However, the sample size was calculated via G*Power. Future studies should include a larger and more diverse population to improve external validity, including the addition of a sham Tuina group. Second, the age of the enrolled children was 16–98 (average, 42.70 ± 17.81) months old. Some results came from their parents due to incomplete comprehension and expression skills. Third, the Tuina in this study was performed by the same skilled therapist, but some variability in the standardization of stimulation intensity by manual manipulation is inevitable. A longer observation period is needed to achieve the long-term efficacy and recurrence rates of constipation in Tui Na.

In conclusion, constipation is a highly prevalent disease of the gastrointestinal tract in children with CP, and Tuina is as effective as medical care in addition to basic treatment. The results of this study suggest that Tui Na may serve as a viable alternative treatment for constipation.

## Data Availability

The raw data supporting the conclusions of this article will be made available by the authors, without undue reservation.

## References

[1] Campanozzi A Capano G Miele E Romano A Scuccimarra G Del Giudice E Impact of malnutrition on gastrointestinal disorders and gross motor abilities in children with cerebral palsy. Brain Dev. (2007) 29:25–9. 10.1016/j.braindev.2006.05.008 16843628

[2] Koloski NA Jones M Wai R Gill RS Byles J Talley NJ. Impact of persistent constipation on health-related quality of life and mortality in older community-dwelling women. Am J Gastroenterol. (2013) 108(7):1152–8. 10.1038/ajg.2013.137 23670115

[3] Veugelers R Benninga MA Calis EAC Willemsen SP Evenhuis H Tibboel D Prevalence and clinical presentation of constipation in children with severe generalized cerebral palsy. Dev Med Child Neurol. (2010) 52:e216–21. 10.1111/j.1469-8749.2010.03701.x 20497454

[4] Park ES Park CI Cho S-R Na S-I Cho YS. Colonic transit time and constipation in children with spastic cerebral palsy. Arch Phys Med Rehabil. (2004) 85:453–6. 10.1016/S0003-9993(03)00479-9 15031832

[5] Jahromi SR Togha M Fesharaki SH Najafi M Moghadam NB Kheradmand JA Gastrointestinal adverse effects of antiepileptic drugs in intractable epileptic patients. Seizure. (2011) 20(4):343–6. 10.1016/j.seizure.2010.12.011 21236703

[6] Vande Velde S Van Renterghem K Van Winkel M De Bruyne R Van Biervliet S. Constipation and fecal incontinence in children with cerebral palsy. Overview of literature and flowchart for a stepwise approach. Acta Gastroenterol Belg. (2018) 81(3):415–8. 30350531

[7] Elawad MA Sullivan PB. Management of constipation in children with disabilities. Dev Med Child Neurol. (2001) 43(12):829–32. 10.1111/j.1469-8749.2001.tb00171.x 11769270

[8] Imanieh MH Golpayegan MR Sedighi M Ahmadi K Aghaie A Dehghani SM Comparison of three therapeutic interventions for chronic constipation in pediatric patients with cerebral palsy: a randomized clinical trial. Prz Gastroenterol. (2019) 14(4):292–7. 10.5114/pg.2019.84872 31988677 PMC6983768

[9] García Contreras AA Vásquez Garibay EM Sánchez Ramírez CA Fafutis Morris M Delgado Rizo V. Lactobacillus reuteri DSM 17938 and Agave inulin in children with cerebral palsy and chronic constipation: a double-blind randomized placebo controlled clinical trial. Nutrients. (2020) 12(10):2971. 10.3390/nu12102971 32998471 PMC7601218

[10] Zhang X Hu L Li L Wang Y Zhang C Su J Pediatric tuina for functional constipation in children: study protocol for a randomized controlled trail. Trials. (2022) 23(1):750. 10.1186/s13063-022-06678-y 36064720 PMC9446667

[11] Longstreth GF Grant Thompson W Chey WD Houghton LA Mearin F Spiller RC. Functional bowel disorders. Gastroenterology. (2006) 130(5):1480–91. 10.1053/j.gastro.2005.11.061 16678561

[12] Heaton KW Lewis SJ. Stool form scale as a useful guide to intestinal transit time. Scand J Gastroenterol. (1997) 32(9):920–4. 10.3109/00365529709011203 9299672

[13] Woolery M Carroll E Fenn E Wieland H Jarosinski P Corey B A constipation assessment scale for use in pediatric oncology. J Pediatr Oncol Nurs. (2006) 23:65–74. 10.1177/1043454205285874 16476780

[14] Hassanein SMA Deifallah SM Bastawy HA. Efficacy of oral magnesium therapy in the treatment of chronic constipation in spastic cerebral palsy children: a randomized controlled trial. World J Pediatr. (2021) 17(1):92–8. 10.1007/s12519-020-00401-0 33481179

[15] Tabbers MM DiLorenzo C Berger MY Faure C Langendam MW Nurko S Evaluation and treatment of functional constipation in infants and children: evidence-based recommendations from ESPGHAN and NASPGHAN. JPGN. (2014) 58(2):258–74. 10.1097/MPG.0000000000000266 24345831

[16] García Contreras AA Vásquez Garibay EM Sánchez Ramírez CA Fafutis Morris M Delgado Rizo V. Factors associated with the stool characteristics of children with cerebral palsy and chronic constipation. Rev Esp Enferm Dig. (2020) 112(1):41–6. 10.17235/reed.2019.6313/2019 31830793

[17] Svedberg LE Englund E Malker H Stener-Victorin E. Parental perception of cold extremities and other accompanying symptoms in children with cerebral palsy. Eur J Paediatr Neurol. (2008) 12(2):89–96. 10.1016/j.ejpn.2007.06.004 17662628

[18] Johanson JF Sonnenberg A Koch TR McCarty DJ. Association of constipation with neurologic diseases. Dig Dis Sci. (1992) 37:179–86. 10.1007/BF01308169 1735333

[19] Fiori S Staudt M Boyd RN Guzzetta A. Neural plasticity after congenital brain lesions. Neural Plast. (2019) 2019:9154282. 10.1155/2019/9154282 31191640 PMC6525879

[20] Sullivan PB. Gastrointestinal disorders in children with neurodevelopmental disabilities. Dev Disabil. (2008) 14:128–36. 10.1002/ddrr.18 18646021

[21] Holey LA Dixon J. Connective tissue manipulation: a review of theory and clinical evidence. J Bodyw Mov Ther. (2014) 18:112–8. 10.1016/j.jbmt.2013.08.003 24411158

[22] Morisawa T Takahashi T Nishi S. Effects of passive lower limb and trunk exercises and diaphragm breathing exercise on intestinal movement. J Phys Ther Sci. (2013) 25(1):117–9. 10.1589/jpts.25.117

[23] Awan WA Masood T. Role of stretching exercises in the management of constipation in spastic cerebral palsy. J Ayub Med Coll Abbottabad. (2016) 28(4):798–801. 28586619

